# A study on the overall variance and void architecture on MEX-PLA tensile properties through printing parameter optimisation

**DOI:** 10.1038/s41598-025-87348-2

**Published:** 2025-01-24

**Authors:** Mirza Faizaan, Satish Shenoy Baloor, Srinivas Nunna, Ravindra Mallya, Sathish Rao Udupi, Chandrakant Ramanath Kini, Sitarama Raju Kada, Claudia Creighton

**Affiliations:** 1 Department of Aeronautical and Automobile Engineering, Manipal Institute of Technology, Manipal Academy of Higher Education, Manipal, Karnataka 576104 India; 2 Institute for Frontier Materials, Deakin University, Waurn Ponds, VIC 3216 Australia

**Keywords:** Fused deposition modeling (FDM), 3D printing, PLA, Tensile properties, Taguchi, Micro-CT, Mechanical properties, Engineering, Imaging techniques

## Abstract

This study investigates the influence of printing parameters on the tensile properties and void architecture of poly(lactic) acid (PLA) parts fabricated using the fused filament fabrication (FFF) technique. Two Taguchi optimisation methods were employed to identify the optimal parameter combinations for maximising tensile performance. The results revealed a positive correlation between tensile performance and nozzle diameter (ND). Notably, the analysis of the coefficient of variance (COV) studies revealed that both tensile strength and modulus exhibited minimal variation within a tight range of 5.5%, suggesting a high degree of process consistency in achieving desired mechanical properties regardless of the chosen parameters within the investigated range. Micro-computed tomography (micro-CT) analysis was used to quantify void characteristics within the as-printed samples. Micro-CT analysis confirmed the influence of ND and layer thickness (LT) on void architecture. By establishing structure–property relationships, the study revealed that larger NDs paired with finer LTs yielded superior tensile properties due to reduced void content. This work highlights the interplay between printing parameters, void architecture, and the resulting mechanical behaviour of MEX-based PLA parts.

## Introduction

Owing to the expiration of fused deposition modelling (FDM) related patents in 2009, the past decade has seen a surge in publications evaluating the effectiveness of printing parameters on the tensile performance of 3D printed parts^1^. The typical literature theme involves varying a set of parameters within a permissible range in the slicing software and validating its effect on the mechanical performance of 3D printed parts. These parameters include but are not limited to layer thickness (LT)^2^, printing speed^2–5^, build orientation^2,6–8^, infill density and pattern^6,9–12^, raster angle^8,9,13–17^, extrusion temperature (ET)^18–21^ and others. The vast majority of the literature finds poly(lactic) acid (PLA) to be the preferred 3D printing material over Acrylonitrile Butadiene Styrene (ABS) for its ease of printing, higher tensile properties and biodegradability^22^. Although it is now confirmed that 0° (longitudinal) rasters with 100% infill density yield the highest tensile performance, conflicting reports still exist on the most suitable parameter combinations to maximise tensile performance.

Many reports confirm low print speed (PS), 100% infill density, higher ET, and 0° raster maximise tensile performance. A meta-analysis of FDM printing parameters on tensile strength identified less heterogeneity when low PS and higher ETs were adopted and recommended the same for higher tensile strength^23,24^. On the other hand, Tymark et al.^4^ noted that PLA specimens showed higher variability up to 22% and 6% for tensile strength and modulus, respectively, with changing LT.

Layer height (LT) is the most studied parameter in literature. While Chacon et al.^2^ Sood et al.^16^ and Alafaghani et al.^25^ agree LT to be a significant contributor to maximise tensile performance, others^3,4,26,27^ in contrast, report LT to have an insignificant contribution to tensile properties. Elaborating on the effect of LT, while some authors^1,16,25,28^ conclude a higher LT maximises tensile performance, citing better homogeneity of tall rasters, others^2,23,29^ conclude a lower LT to be beneficial, citing smaller air gaps between adjacent rasters. The reporting of low and high LT is again arbitrary, wherein Lanzotti et al.^1^ reports 0.2 mm as a high LT, while Christiyan et al.^30^ refer to 0.2 mm as a low LT. However, 0.2 mm is in the nominal printing range, with 0.1 mm and 0.3 mm being the permissible extremes for a 0.4 mm nozzle diameter (ND) used in these studies. Although LT defines the height of each raster in consecutive layers, the effect of raster width through ND is under-reported in the literature.

Triyono et al.^31^ evaluated the effect of nozzle size ranging from 0.3 to 0.6 mm for samples printed with LT at 20% nozzle size. They confirmed that bigger nozzles improve the density and tensile performance of the parts. Corral et al.^11^ compared the pore size within printed specimens when using 0.2 mm and 0.4 mm nozzles and found an increase in pore size with the 0.4 mm nozzle while the overall porosity remained the same. Studies on the effect of ND on the melt flow behaviour shed some light on the efficacy of larger nozzles to print consistent rasters^32^. Wang et al.^33^ provided the optimal printing parameters for polyetheretherketone (PEEK) using three NDs 0.4, 0.6, and 0.8 mm and concluded lower PS and LT to maximise tensile performance. However, a comparison between different NDs was not evaluated in the study. Kinski et al.^34^ provided insights on improved sample breaking force with increasing ND for samples with less than 100% infill. Several studies confirmed increased tensile strength and modulus with increasing ND^22,31,35^. However, none of the above studies compared the effect of ND in conjunction with varying LT.

Micro-CT has been employed in recent literature to characterise the pore size and distribution in 3D printed parts. The analysis revealed spherical and elongated pores within the 3D printed structures, where spherical pores embedded in the intra-layers and elongated voids were observed between insufficiently fused layers^36^. Gendviliene et al.^37^ raised concerns about the precision of micro-CT for quantifying void volume. They reported that the scanned volume was significantly larger than the actual model and was influenced by its resolution. Despite this limitation, micro-CT is reliable for comparative morphological analysis. The method identified a stretching effect and filament widening at material crossovers, contributing to pore formation^38^, elucidating that the pores originated from abrupt changes in the nozzle trajectory and are mainly influenced by the printing speed^39^. Further, it was reported that pores are homogeneously distributed, with larger pores predominantly located at the shell walls forming a grid-like structure and limited in-plane pore continuity for ± 45° infills^39–41^. Nonetheless, the overall porosity in the bulk of the material was reduced with consecutive layers (layering effect), and the highest porosity was observed for the top layer^39^.

This study aims to comprehensively understand the extent of variance in tensile performance when printing parameters are varied. Two Taguchi L9 orthogonal array experiments were carried out to study the effect of printing parameters on raster geometry and stacking and, subsequently, on the tensile properties of FDM-PLA coupons. This paper optimises with varying ND, LT and ET in the first Taguchi study, followed by a second optimisation with LT, PS and the number of ± 45° top/bottom layers. The results are statistically optimised to maximise the tensile strength and modulus of FDM-PLA. A perspective on the extent of variability within the test coupons and across the entire sample dataset is presented using the coefficient of variance (COV) for both strength and modulus for varying printing parameter combinations. Further, structure–property relationships for ND and LT on void fraction and void size is evaluated using micro-CT and ImageJ software was used to quantify voids from micro-CT’s 2D slices.

## Materials and methods

### PLA filaments and 3D printing

A commercially available 1.75 mm eSun PLA + white filament was used to 3D print 6 mm thick ASTM D638 Type I tensile dog-bone specimens on an open-source Creality Ender 3 V2 3D printer. All specimens were printed flat at a bed temperature of 60 °C. To ensure consistent behaviour, a 100% flow, 100% infill density, and a concentric infill pattern were employed. Two printing conditions were tested: Taguchi #1, with two zig–zag top/bottom layers printed at a speed of 60 mm/s, and Taguchi #2, using a 0.6 mm nozzle at an extrusion temperature of 210 °C.

### Taguchi analysis

The Taguchi L9 analysis was carried out on the Minitab 19 statistical software using the design of experiments (DOE) methodology for Taguchi, and optimised parameters were statistically obtained to maximise tensile strength and modulus. Two Taguchi L9 DOE were adopted to evaluate the effects of three printing parameters in each case, as tabulated in Table 1, followed by a summary of the printing parameter combinations per Taguchi L9 DOE.

**Table 1:** Summary of Taguchi L9 DOE for printing parameter evaluation.

	Parameter			Level 1	Level 2	Level 3
Taguchi L9 #1	Nozzle Diameter [ND] (mm)			0.4	0.6	0.8
	Layer Thickness [LT] (mm)			0.12	0.15	0.2
	Extrusion Temperature [ET] (°C)			195	205	215
Taguchi L9 #2	Layer Thickness [LT] (mm)			0.15	0.24	0.3
	Print Speed [PS] (mm/s)			20	60	100
	No. Top/Bottom layers [TB]			0	2	4

Experiment #	Taguchi #1			Taguchi #2		
	ND	LT	ET	LT	PS	TB
1	0.4	0.12	195	0.15	20	0
2	0.4	0.15	205	0.15	60	2
3	0.4	0.20	215	0.15	100	4
4	0.6	0.12	205	0.24	20	2
5	0.6	0.15	215	0.24	60	4
6	0.6	0.20	195	0.24	100	0
7	0.8	0.12	215	0.30	20	4
8	0.8	0.15	195	0.30	60	0
9	0.8	0.20	205	0.30	100	2

### Mechanical testing

**Fig. 1:**
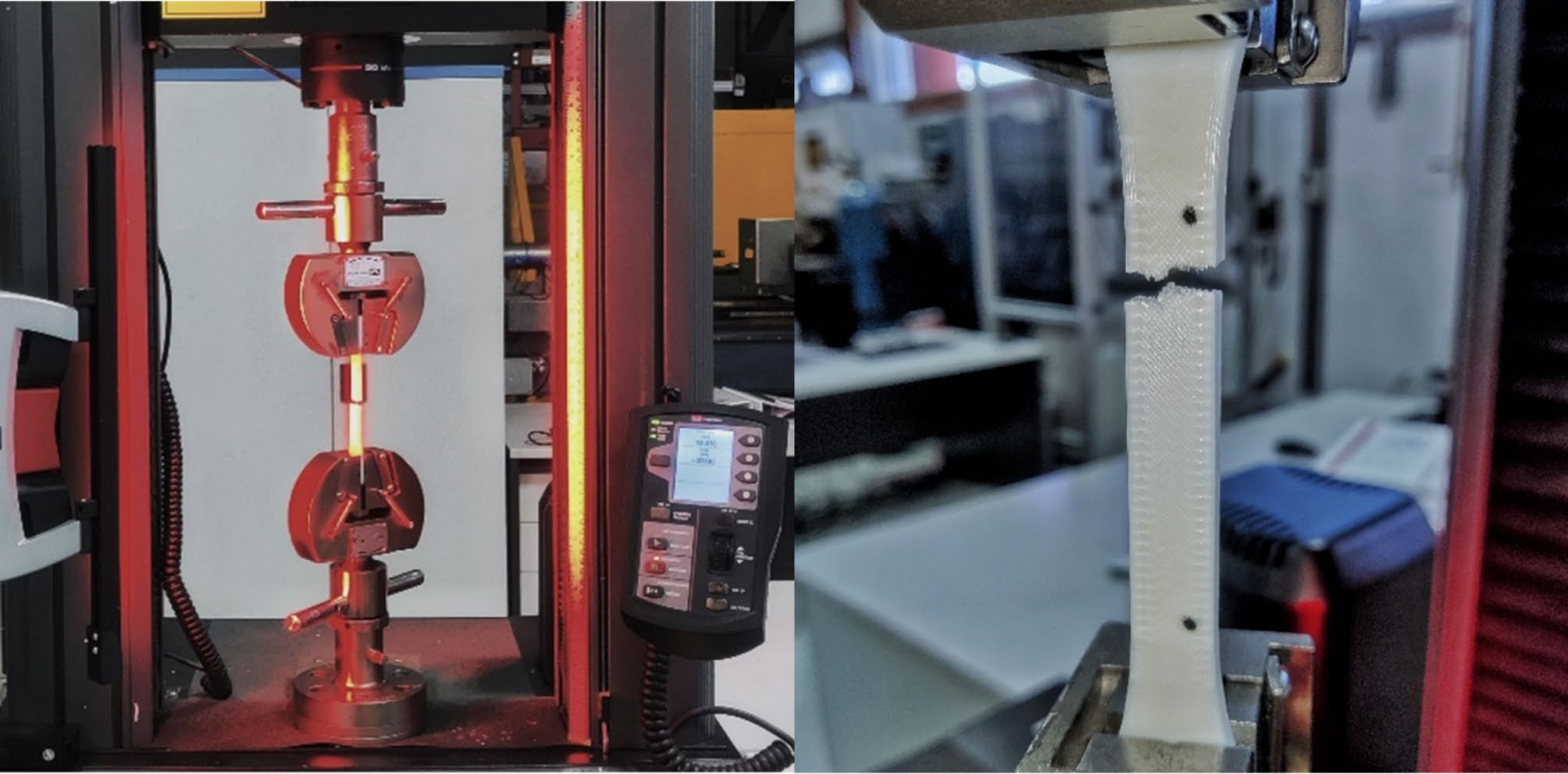
The Instron 30KN UTM equipped with a video extensometer tensile testing setup for the 6 mm thick ASTM Type-1 dog-bone coupon fabricated for the optimisation studies.

Tensile tests were performed on the Instron 30KN UTM with a 5 mm/min testing speed, and strain measurements were recorded using a video extensometer as seen in Fig. 1. Five samples of each parameter combination were tested for consistency.

### Microstructural analysis

#### SEM

Scanning electron microscope images of the fractured tensile coupons were captured on a ZEISS EVO MA18 with Oxford EDS(X-act). SEM images were captured only for the 0.6 mm ND with varying layer heights (0.15, 0.24, and 0.30 mm) to understand the fracture mechanisms.

#### Micro CT

Micro-CT scans were captured for as-printed rectangular bar samples (12 × 12 × 24 mm) with varying ND and LT to replicate the tensile coupon gauge length. Five samples with varying ND (0.8, 0.6, 0.4 mm) printed at a constant 0.15 mm LT and varying LT (0.15, 0.24, 0.3 mm) printed with the 0.6 mm ND were scanned. The X-Ray projection images were captured using a tungsten x-ray source with an accelerating voltage of 50 kVP and a 2W tube power with an integration time of 7 s using. Approximately 720 X-ray projection images were collected during 360° sample rotation with an effective pixel size of 2.89 (± 0.087)µm. The cross-sectional slices were obtained by reconstruction using X-TRACT software package. Lastly, Avizo 2024.1 software was used for 3D visualisation and analysis.

#### Void analysis

ImageJ was used to identify voids by selecting a 5 × 5 mm region of interest (ROI) at the centre of each of the 2D micro-CT slices for each specimen with varying NDs and LTs. The 2D slices for each specimen were stacked in ImageJ to mimic a 5 × 5 × 5 mm cube to quantify the void sizes across the sample volume, and the void area fraction on each slice was averaged. Void areas were identified by local thresholding, and the structure–property relationships were analysed using MATLAB.

## Results

### Tensile performance

A 16.21–30.78% decrement in filament tensile strength of 63MPa^42^ was observed in the test coupons, possibly owing to characteristic process-induced defects. The tensile performance of Taguchi #1 and Taguchi #2 are tabulated in Table 2. Interestingly, predominantly horizontal trends were observed for both tensile strength and modulus, with larger nozzles and slower speeds resulting in better tensile performance. The highest tensile strength was observed for the combination 0.8 mm ND, 0.15 mm LT and 195 °C ET, while the highest modulus was obtained for 0.8 mm ND, 0.12 mm LT and 215 °C ET in the case of Taguchi #1. Similarly, for specimens printed with a 0.6 mm nozzle in Taguchi #2, the highest strength and modulus were obtained for the combinations, 0.3 mm LT, 60 mm/s PS, and 0 TB, and 0.15 mm LT 20 mm/s PS and 0 TB, respectively. Analysis of variance (ANOVA) optimisations were carried out on the tensile results to optimise for maximum strength and modulus and main effects plots for signal–noise (SN) ratios were calculated.

**Table 2:** Tensile performance of Taguchi #1 (constant PS–60 mm/s) and Taguchi #2 (constant ND – 0.6 mm).

#	Taguchi #1				Taguchi #2			
	Tensile strength [MPa]		Tensile modulus [MPa]		Tensile strength [MPa]		Tensile modulus [MPa]	
1	46.36	± 0.54	3051.26	± 31.13	48.91	± 0.87	3409.07	± 218.67
2	49.00	± 0.57	3085.33	± 66.02	47.91	± 0.37	3123.35	± 156.83
3	46.03	± 2.19	3092.16	± 81.31	46.19	± 0.45	3027.43	± 58.60
4	49.74	± 0.72	3159.42	± 57.94	47.53	± 0.27	3348.08	± 14.42
5	50.67	± 0.15	3142.08	± 23.42	46.19	± 0.65	3097.08	± 61.26
6	49.90	± 0.61	3123.67	± 25.71	45.83	± 0.36	3006.01	± 240.96
7	52.08	± 0.51	3431.40	± 76.70	44.65	± 0.41	3110.02	± 119.81
8	52.79	± 0.45	3352.74	± 98.14	49.50	± 0.38	3109.42	± 104.74
9	52.00	± 0.60	3382.31	± 124.25	43.60	± 0.51	2762.01	± 78.11

#### Taguchi #1

**Fig. 2:**
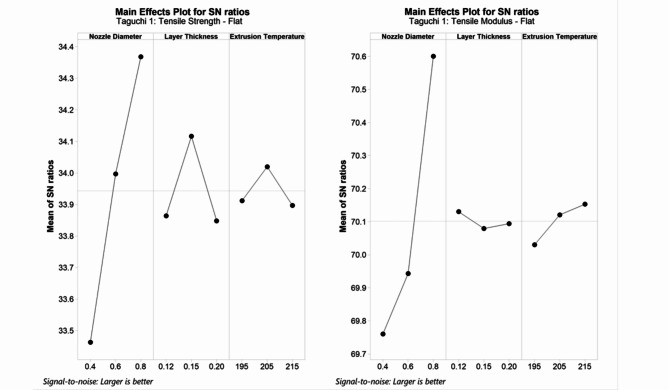
Main effects plot for SN ratios—Taguchi #1.

Taguchi optimisation was carried out to evaluate the optimal parameter combination to maximise tensile strength and modulus, respectively. Table 3 summarises the ANOVA for tensile strength and modulus, with both models indicating a good fit with an R-Sq > 97%. It is clear from the ANOVA results that only ND is a significant factor influencing tensile strength (*p* = 0.03) and modulus (*p* = 0.004) with 86.08% and 97.24% contributions, respectively. Comparing the F and *p* values, ND affects tensile modulus much more than strength. LT and ET do not significantly influence strength or modulus response for flat-oriented samples as confirmed from their *p* > 0.05. Summarising from the main effects plot for SN ratios in Fig. 2, the optimal parameters to maximise tensile strength and modulus for flat orientation would be printed with a 0.8 mm ND. 0.15 mm and 0.12 mm LT, 205 °C and 215 °C ET are preferred for tensile strength and modulus, respectively.

**Table 3:** Summary of ANOVA for tensile strength and modulus—Taguchi #1.

Tensile Strength							
S	0.1385						
R-Sq	97.34%						
R-Sq(adj)	89.38%						

Source	DF	Seq SS	Adj SS	Adj MS	F	*p*	Contribution
Nozzle Diameter	2	1.24331	1.24331	0.62166	32.42	**0.030**	86.08%
Layer Thickness	2	0.13578	0.13578	0.06789	3.54	0.220	9.40%
Extrusion Temperature	2	0.02693	0.02693	0.01347	0.70	0.587	1.86%
Residual Error	2	0.03835	0.03835	0.01918			2.66%
Total	8	1.44437					

Tensile Modulus							
S	0.0492						
R-Sq	99.60%						
R-Sq(adj)	98.39%						

Source	DF	Seq SS	Adj SS	Adj MS	F	*p*	Contribution
Nozzle diameter	2	1.17158	1.17158	0.585789	241.58	**0.004**	97.24%
Layer thickness	2	0.00412	0.00412	0.002058	0.85	0.541	0.34%
Extrusion temperature	2	0.02429	0.02429	0.012144	5.01	0.166	2.02%
Residual error	2	0.00485	0.00485	0.002425			0.40%
Total	8	1.20483					

Significant values are in [bold].

#### Taguchi #2

In the second Taguchi study, LT, PS and the number of ± 45° top/bottom layers were evaluated for tensile coupons printed with a 0.6 mm nozzle diameter. These parameters control the number of layers, the stretch on the extrudate and the combination of 0° and ± 45° rasters in the loading axis. The summary of the Taguchi analysis for tensile results is summarised in Table 4. The *p*-values obtained indicate that neither parameter significantly influences tensile performance. The poor R-Sq (adj) for tensile strength at 34.89% indicates a poor fit for the model. However, we can conclude from the F values that PS affects tensile performance more than LT and the number of 45° top/bottom layers.

**Table 4:** Summary of ANOVA for tensile strength and modulus—Taguchi #2.

Tensile strength							
S	0.2913						
R-Sq	83.72%						
R-Sq(adj)	34.89%						

Source	DF	Seq SS	Adj SS	Adj MS	F	p	Contribution
Layer thickness	2	0.1735	0.1735	0.08674	1.02	0.495	16.64%
Print speed	2	0.3861	0.3861	0.19307	2.27	0.305	37.02%
No. TB layers	2	0.3135	0.3135	0.15676	1.85	0.351	30.06%
Residual error	2	0.1698	0.1698	0.08488			16.28%
Total	8	1.0429					

Tensile modulus							
S	0.2231						
R-Sq	95.58%						
R-Sq(adj)	82.32%						

Source	DF	Seq SS	Adj SS	Adj MS	F	p	Contribution
Layer thickness	2	0.5058	0.5058	0.2529	5.08	0.164	22.46%
Print speed	2	1.49889	1.49889	0.74945	15.06	0.062	66.56%
No. TB layers	2	0.1476	0.1476	0.0738	1.48	0.403	6.55%
Residual error	2	0.09952	0.09952	0.04976			4.42%
Total	8	2.25182					

**Fig. 3:**
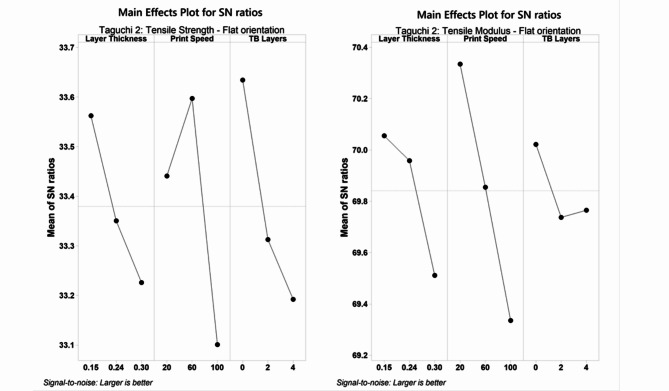
Main effects plot for SN ratios—Taguchi #2.

Although the selected parameters are insignificant in maximising tensile properties, the optimal parameters from the main effects plot for SN ratios (Fig. 3) recommend a finer layer thickness of 0.15 mm to maximise both tensile strength and modulus. A nominal PS at 60 mm/s is suggested for higher strength, and a slower PS at 20 mm/s to maximise modulus. The effect of raster angle is evident in this case study as the main effects plot for both strength and modulus recommend all rasters to be in loading axis direction with no 45° top/bottom layers.

### Imaging

#### SEM micrographs

**Fig. 4:**
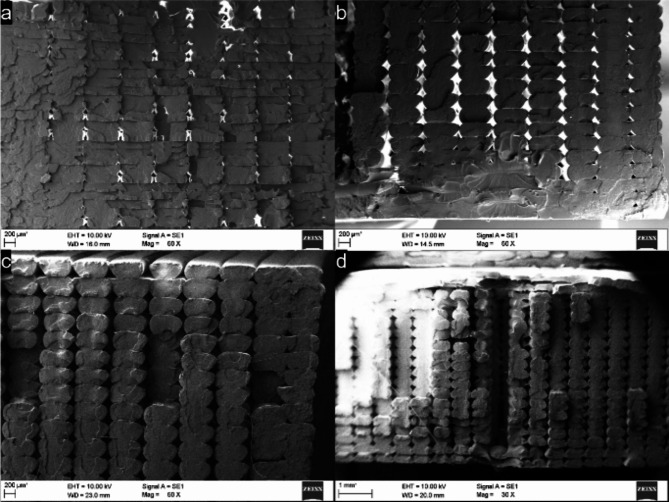
SEM micrographs of tensile fracture surfaces for samples printed with 0.6 mm ND. (**a**–**c**) show samples printed with 0.15 mm, 0.24, and 0.3 mm LT, respectively. (**d**) highlights the interlayer void formed due to the concentric infill pattern at the neck region of the dog-bone sample.

The SEM images of the fractured surfaces reveal predominantly brittle fractures in the specimens, irrespective of the varying LT. It can be noted in Fig. 4a–c that the raster cross-section gradually changes from rectangular to oval as the layer height increases. Squishing of the bottom layers was also observed with higher LTs, while rasters in the bottom layers of samples printed with 0.15 mm LT show more homogenous intralayer adhesion, especially at the walls. River markings observed in samples printed with 0.15 mm LT (a) indicate a combination of interlayer and intralayer fracture propagation. In comparison, fracture propagation along consecutive layers was observed for the 0.3 mm LT (c).

As the concentric infill pattern follows the dog-bone profile from the perimeter to the centre, it tends to leave small gaps at the neck region where the concentric infill takes a u-turn after the gauge section is filled, leaving a gap that runs across all layers. Figure 4d shows the SEM image captured at 30 × magnification of the samples fractured at the neck region originating from the void at the centre of the sample. Nonetheless, the tensile properties of the resulting coupons yielded values similar to the results reported in literature^4^.

#### Micro CT

**Fig. 5:**
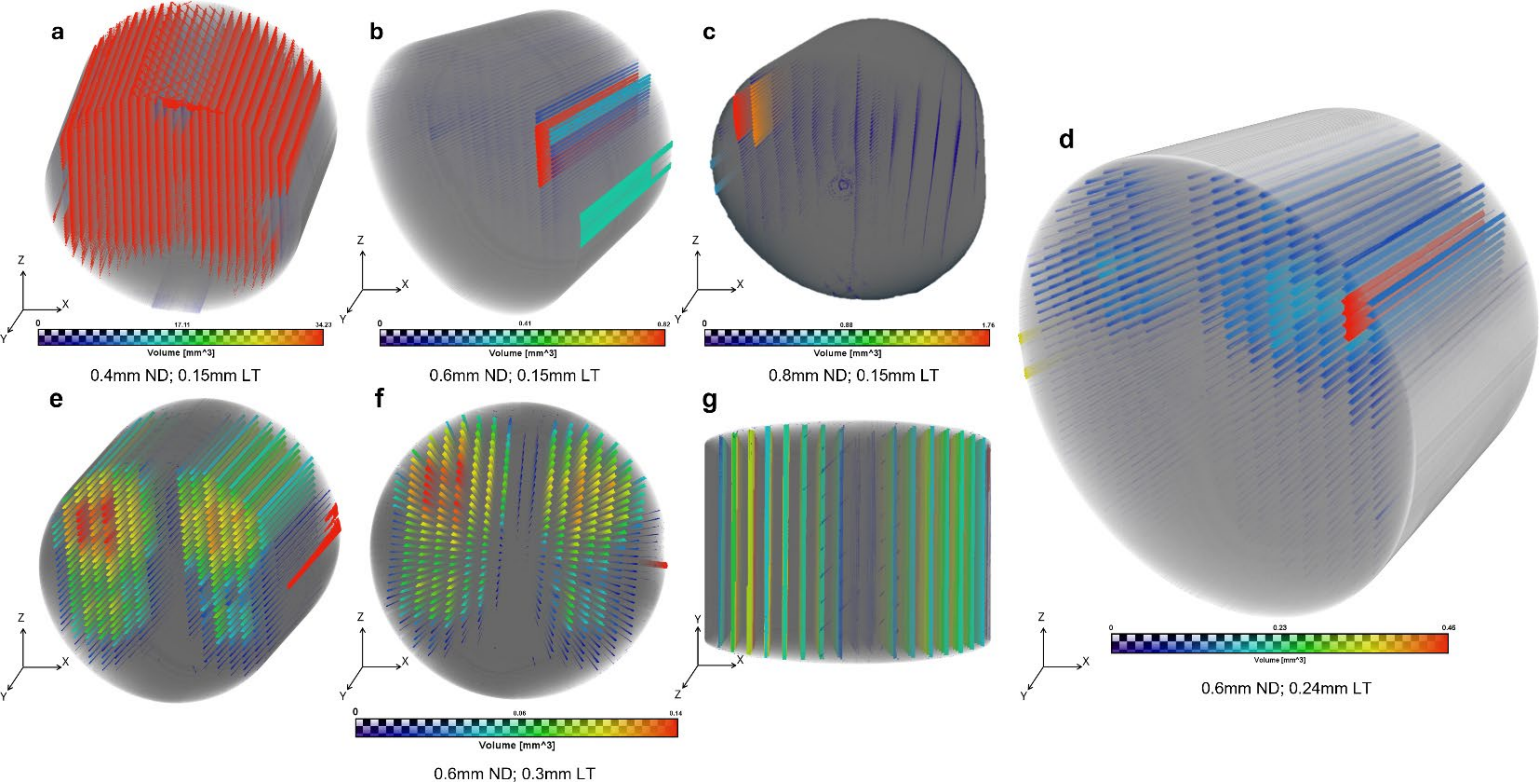
3D reconstruction of micro-CT scans for a rectangular bar sample with varying ND and LT. (**a**–**c**) Samples printed with a 0.15 mm LT and varying ND, namely, 0.4, 0.6 and 0.8 mm, respectively. (**d**) Distribution of voids in samples printed with 0.6 mm ND and 0.24 mm LT in isometric view. (**e**–**g**) distribution of voids for samples printed with 0.6 mm ND and 0.3 mm LT in isometric, front and side views, respectively.

The 2D scans captured along the gauge length were reconstructed to a 3D volume (12 × 12 × 24 mm) using Avizo software. In the 3D model, the voids were colour-coded by size to highlight the void size distribution with varying ND and LT. In line with previous reports^36^, it can be seen in the 3D reconstructions (Fig. 5a–c) that the size of voids is homogeneously and periodically distributed in the samples. The voids are smallest at the bottom of the specimens, and the rasters are conjoined with no air gaps. The voids grow gradually from the bottom and centre of the specimens towards the walls, with the largest voids observed at the top and edges of the printed samples. Figure 5d–g shows micro-CT reconstructions for samples printed with constant ND of 0.6 mm and varying LTs, namely 0.24 mm (Fig. 5d) and 0.3 mm (Fig. 5e–g).

Similar to samples printed with varying ND at constant LT, it is evident from these images that the concentric infill pattern with 100% density renders a homogenous phase at the bottom centre of the specimen, and the voids grow towards the walls and across consecutive layers. The voids are axially connected along the y-direction for all the reconstructions, as was also reported by Guessasma et al. in their study^39^. It is confirmed from these 3D reconstructions that the characteristic FDM process-induced voids follow the toolpath and raster orientation throughout the 3D printed part but are nearly void-free at the bottom layers as they get squished with consecutive layers. Future studies may compare 100% solid infill patterns other than *“concentric infill”* to understand the void distribution and the layering effect on the voids for the first few layers.

### Void analysis

**Fig. 6:**
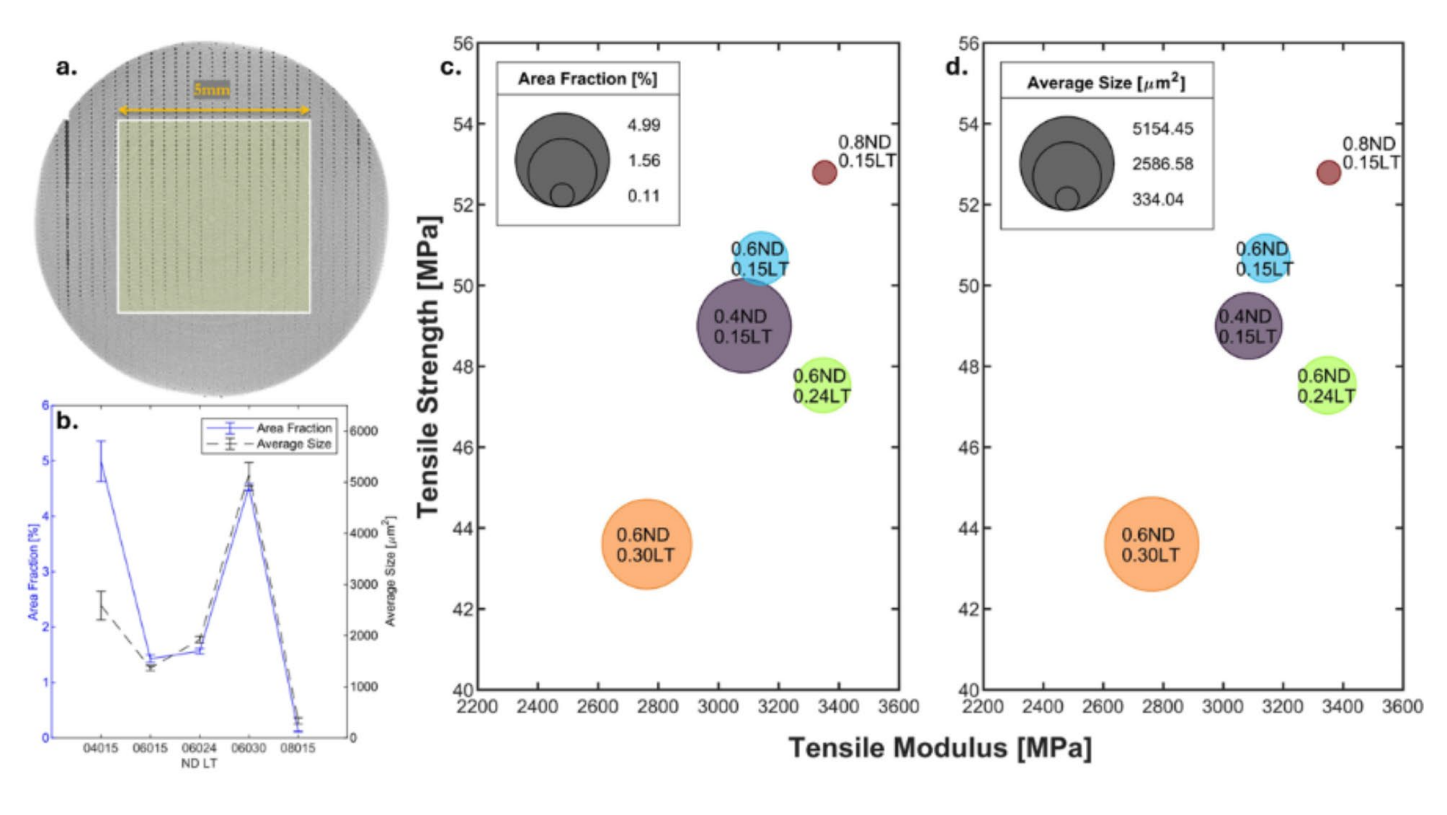
(**a**) A 5 × 5 mm ROI selected for local thresholding in ImageJ. (**b**) Area fraction and average void size of the air gaps quantified for varying ND at constant LT and varying LT at constant ND. (**c** and **d**) Structure–Property relationship of tensile performance vs. area fraction and average size of voids, respectively.

Micro-CT analysis was employed to quantify void architecture within the additively manufactured samples. Local thresholding was performed on ImageJ software (National Institutes of Health, Maryland, USA) and segmented the micro-CT slices, enabling the determination of average area fraction and void size. A representative 3D printed cube (5 mm × 5 mm × 5 mm) was analysed by selecting a 5 mm × 5 mm region of interest (ROI) across a 5 mm depth (z-distance) on each slice (Fig. 6a). The average area fraction for each slice and the mean void size were subsequently calculated and plotted in Fig. 6b.

The results revealed a clear trend, indicating an inverse correlation between ND and void properties. Conversely, a linear relationship was observed between ND and void architecture. Like the results by Guessasma et al.^39^, who quantified the axial porosity of 3D printed parts to reach 5.73%, the void area fraction for varying ND and LT was observed to range between 0.117 and 4.99% of the total printed cross-section. The void size peaked at 5154.45 ± 231.45 µm^2^ for samples printed with 0.6 mm ND and 0.3 mm LT, while the smallest voids were observed in samples printed with 0.8 mm ND and 0.15 mm LT at 334.04 ± 60.51 µm^2^.

The influence of void architecture on tensile properties was investigated by correlating void area fraction and average void size with tensile strength and modulus (Fig. 6c and d, respectively). The data suggest that larger NDs paired with finer LT led to superior tensile properties. At a constant LT of 0.15 mm, both tensile strength and modulus improved with increasing ND. Interestingly, at a fixed ND of 0.6 mm, thicker layers significantly decreased tensile strength compared to 0.15 mm LT. Similarly, the tensile modulus also showed a significant decline for 0.3 mm LT, while contrastingly, 0.24 mm LT resulted in the highest tensile modulus for samples printed with 0.6 mm ND.

## Discussion

**Fig. 7:**
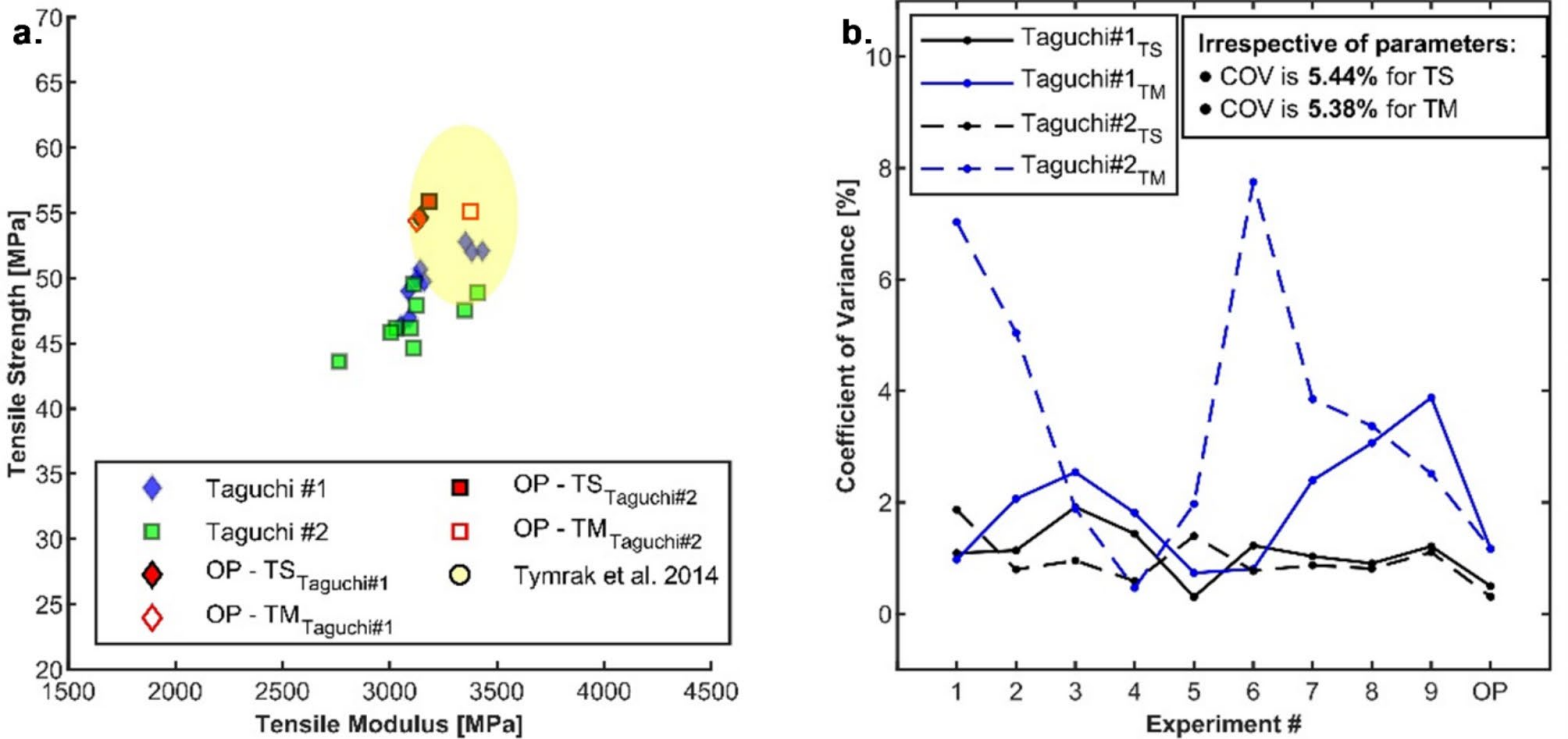
(**a**) Tensile performance and (**b**) Coefficient of Variance (COV %) of optimised FDM PLA flat-oriented coupons for Taguchi #1 and Taguchi #2. OP—Optimised parameter.

This study employed a Taguchi optimisation approach to maximise the tensile performance of AM parts. The results of the confirmation experiments realised through applying optimised parameter (OP) settings are provided in Fig. 7a. A successful improvement in tensile strength was observed with the optimisation. OP parameter combination consistently yielded higher tensile strength than other combinations within each Taguchi study, demonstrating the effectiveness of the optimisation process. However, the impact of optimisation on the modulus of elasticity presented a more nominal outcome.

While optimisation did not significantly alter the overall variability of the modulus, a closer examination revealed study-specific differences. In Taguchi #2, the OP settings improved tensile strength and modulus, indicating a successful optimisation strategy in achieving both desired properties simultaneously within this specific study. Conversely, Taguchi 1 demonstrated minimal change in modulus at the OP point, suggesting a potential benefit not directly captured in this analysis, such as faster printing speeds or reduced material usage.

The yellow ellipse included in the cluster plot (Fig. 7a) represents the extent of tensile strength and modulus from the optimisation study conducted by Tymrak et al.^4^. Despite utilising a distinct parameter set different from ours, the study’s reported tensile performance falls within a narrow range of tensile strength and modulus, like the observations made in this study. Similarly, Popovic et al.^43^ observed that the variation in ultimate tensile strength (UTS) of PLA was less pronounced for samples printed with extrusion temperatures above 190 °C to 210 °C, irrespective of the printing speeds ranging from 53 to 57 MPa. However, the standard deviations were not reported and could be used to examine the variation within the sample types. These reports suggest that achieving near mean mechanical properties might be possible through various printing parameter combinations, offering flexibility in the printing process and providing valuable insights into alternative approaches to achieving the desired tensile performance.

The analysis of the COV shown in Fig. 7b revealed a vital strength of the printing process—its stability. The observed low COV values of < 2% for tensile strength and < 8% for modulus across individual settings for both Taguchi studies highlight high consistency in achieving the desired mechanical properties. Further, combining the tensile properties of both studies, the COV is a meagre 5.44% and 5.38% for tensile strength and modulus, respectively. It can be stated that the tensile strength and modulus may fall within 5.5% of the nominal range regardless of the chosen printing parameters within the investigated range. The variance in tensile performance can be attributed to the characteristic process-induced voids and defects evident with MEX.

It is important to acknowledge potential limitations associated with the chosen parameter ranges. The selection of upper and lower limits for each investigated parameter might have limited the observed variation in tensile performance. A more comprehensive range of parameter exploration could potentially reveal a prominent influence on tensile properties. Future studies employing broader parameter bounds are recommended to obtain a more comprehensive understanding of how printing parameters influence the tensile strength and modulus of AM parts.

## Conclusion

This study investigated the interplay between printing parameters, void architecture and the resulting tensile properties of additively manufactured parts. The analysis revealed a positive correlation between tensile performance and ND, suggesting improved tensile strength and modulus for samples printed with larger nozzle orifice. A perspective on the overall variance was calculated using COV for varying parameter combination and within the same sample set. The robustness of the process to achieve consistent tensile properties was evident across varying processing conditions, as indicated by the low COV for both tensile strength and modulus, remaining below 5.5% irrespective of the chosen printing parameters.

Micro-CT analysis confirmed the influence of ND and LT on void architecture and distribution. An inverse correlation was observed between ND and void properties, while LT demonstrated a linear relationship with void characteristics. Establishing the structure–property relationships revealed that larger NDs paired with finer LTs yielded superior tensile properties attributed to the reduced void size and content achieved with these parameter combinations.

## Data Availability

The authors declare that the data supporting the findings of this study are available within the article. The generated datasets are also available from the first and the corresponding authors on reasonable request.
